# Crosstalk between SDF-1/CXCR4 and SDF-1/CXCR7 in cardiac stem cell migration

**DOI:** 10.1038/srep16813

**Published:** 2015-11-18

**Authors:** Dong Chen, Yanli Xia, Ke Zuo, Ying Wang, Shiying Zhang, Dong Kuang, Yaqi Duan, Xia Zhao, Guoping Wang

**Affiliations:** 1 Institute of Pathology, Tongji Hospital, Tongji Medical College, Huazhong University of Science and Technology, Wuhan 430030, China

## Abstract

Stromal cell-derived factor 1 (SDF-1) is a chemokine that can be expressed in injured cardiomyocytes after myocardial infarction (MI). By combining with its receptor CXCR4, SDF-1 induced stem and progenitor cells migration. CXCR7, a novel receptor for SDF-1, has been identified recently. We aimed to explore the roles of SDF-1/CXCR4 and SDF-1/CXCR7 pathway and their crosstalk in CSCs migration. In the present study, CXCR4 and CXCR7 expression were identified in CSCs. Transwell assay showed that SDF-1 caused CSCs migration in a dose- and time-dependent manner, which could be significantly suppressed by CXCR4 or CXCR7 siRNA. Phospho-ERK, phospho-Akt and Raf-1 significantly elevated in CSCs with SDF-1 stimulation. Knockdown of CXCR4 or CXCR7 significantly decreased phospho-ERK or phospho-Akt, respectively, and eventually resulted in the inhibition of CSCs migration. Moreover, western blot showed that MK2206 (Akt inhibitor) increased the expression of phospho-ERK and Raf-1, whereas PD98059 (ERK inhibitor) had no effect on phospho-Akt and Raf-1. GW5074 (Raf-1 inhibitor) upregulated the expression of phospho-ERK, but had no effect on phospho-Akt. The present study indicated that SDF-1/CXCR7/Akt and SDF-1/CXCR4/ERK pathway played important roles in CSCs migration. Akt phosphorylation inhibited Raf-1 activity, which in turn dephosphorylated ERK and negatively regulated CSCs migration.

Myocardial infarction (MI) remains an important cause of mortality by ultimately leading to heart failure or sudden cardiac death. Increasing evidence indicate that recruitment or direct injection of stem or progenitor cells could participate in the regeneration of heart tissue and improve cardiac function^1^,^2^,^3^. Cardiac stem cells (CSCs) are a pure population of cloned c-kit^+^ cells that reside mainly in the atrium-ventricle groove (AV-groove)^4^. They can be attracted to injured myocardium and differentiated into cardiomyocytes, smooth muscle cells and vascular endothelium. CSCs have been proved to have a therapeutic potential for limiting infarct size and restoring the cardiac function after irreversible ischemic injury^1^,^2^,^5^. Despite these encouraging observations, the mechanism of CSCs to repair the heart remains unclear. Especially, how do CSCs migrate into the peri-infarcted areas after MI occurs?Stromal cell-derived factor 1 (SDF-1), also known as CXCL12, is a kind of chemokine with highly conserved isoforms^6^. It is expressed by a variety of tissues including the bone marrow, heart, liver, lung, lymph nodes, brain, kidney, and pituitary^7^. Recent genome-wide association studies (GWAS) revealed SDF-1 as an important candidate gene associated with coronary artery disease (CAD) and MI, but the underlying mechanisms remain totally unclear^8^,^9^,^10^,^11^,^12^.

The chemokine receptor CXCR4 belongs to the family of seven-span transmembrane G-protein-coupled chemokine receptors (GPCRs). CXCR4 and its ligand SDF-1 are mostly studied for their crucial roles in the homing of stem and progenitor cells in the bone marrow, chemotaxis, cell arrest, angiogenesis, metastasis and cell survival^13^. For a long time, CXCR4 was thought to be the only receptor for SDF-1, with SDF-1 being its only ligand.

In 2005, SDF-1 was revealed to bind a second chemokine receptor CXCR7 with an even 10-fold higher affinity compared with CXCR4^14^. CXCR7 was found to be associated with proliferation, apoptosis, migration, invasion and tumor growth^15^,^16^,^17^. In different cell types, CXCR4 and CXCR7 may be expressed uniquely or in combination. When co-expressed, CXCR4 and CXCR7 may form homo- and heterodimers. CXCR4-CXCR7 heterodimerization especially seems to play an important role in the modulation of the down-stream signaling^18^,^19^,^20^. However, it is largely unknown about the roles of SDF-1/CXCR4 and SDF-1/CXCR7 pathway in cardiac stem cells (CSCs) migration.

Akt, a serine/threonine protein kinase, is a critical downstream target of phosphatidylinositol 3-kinase (PI3K) and plays an important role in regulating cellular functions. Growing evidence indicated that activation of Akt regulated cell migration^21^,^22^,^23^. Equally, extracellular signal-regulated kinase (ERK), a member of mitogen-activated protein kinases (MAPKs) family, can transduce a large number of extracellular information into intracellular responses. ERK signaling pathway has also been reported to play an important role in cell chemotactic response^24^,^25^. Recent reports indicated that CXCR7 can activate Akt, MAPK, and JAK/STAT3 cascades, either by direct modulation, through a β-arrest independent pathway^26^,^27^,^28^,^29^, or after heterodimerization with CXCR4^18^,^20^,^28^,^30^.

Based on these findings, the aim of the present study was to elucidate the role of SDF-1/CXCR4 and SDF-1/CXCR7 pathway in regulating CSCs migration and explore the crosstalk of the signal cascades involving in it.

## Results

### Identification of CSCs and detection of CXCR4/CXCR7

**Figure 1:**
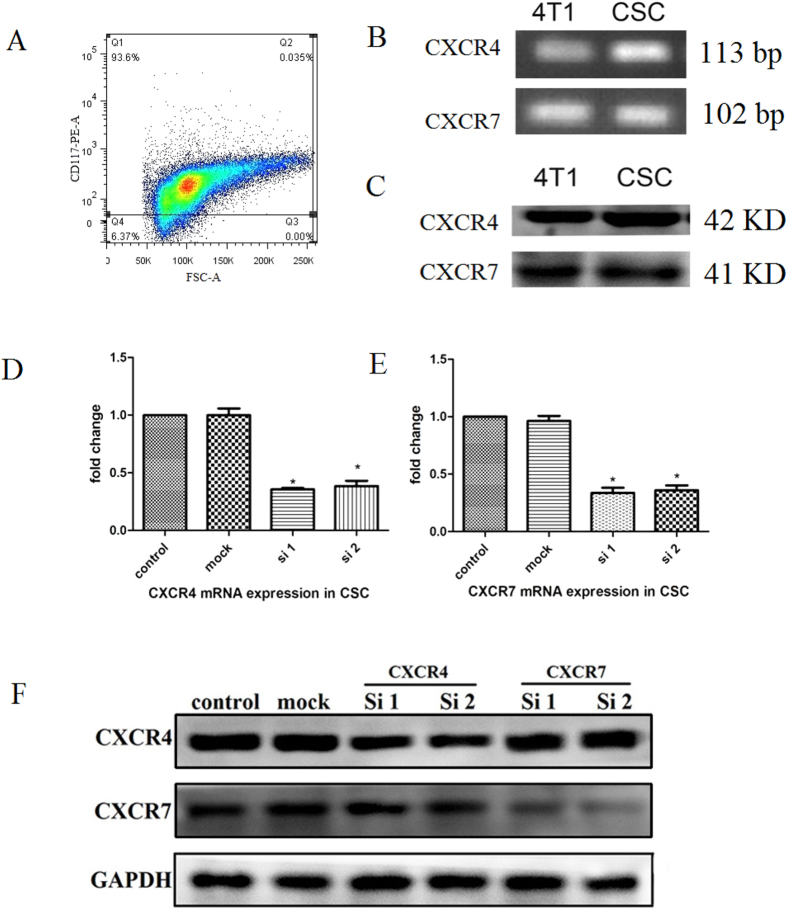
Identification of CSCs and detection of CXCR4 and CXCR7 in CSCs. (**A**) Identification of c-kit^+^ CSCs with a purity of 93.6% by flow cytometry. (**B**) RT-PCR analysis of CXCR4 and CXCR7 mRNA in CSCs. 4T1 cells were used as a positive control. (**C**) Western blot analysis of CXCR4 and CXCR7 protein in CSCs. 4T1 cells were used as a positive control. (**D**) RT-PCR analysis of CXCR4 mRNA in CSCs with CXCR4 siRNA. (**E**) RT-PCR analysis of CXCR7 mRNA in CSCs with CXCR7 siRNA. (**F**) Western blot analysis of CXCR4 and CXCR7 protein in CSCs with CXCR4 siRNA and CXCR7 siRNA. *P < 0.05 versus control.

The purity of c-kit^+^ CSCs was 93.6% as determined by flow cytometry (Fig. 1A). The expressions of CXCR4 and CXCR7 in CSCs were determined by RT-PCR and western blot in which 4T1 cells were treated as a positive control (Fig. 1B,C). After specific siRNA transfection in CSCs, the mRNA levels of CXCR4 and CXCR7 significantly reduced as shown in Fig. 1D,E. Protein levels of them were shown in Fig. 1F. All these results indicated that CXCR4 and CXCR7 could be detected in CSCs.

### Effects of SDF-1, CXCR4 and CXCR7 on CSCs migration *in vitro*

**Figure 2:**
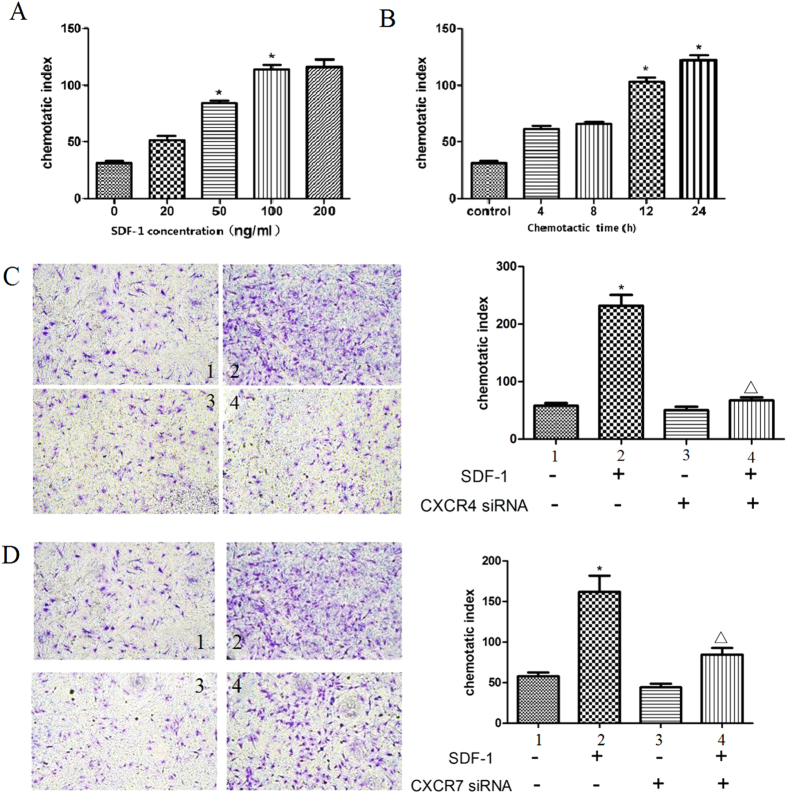
Effects of SDF-1, CXCR4 and CXCR7 on CSCs migration *in vitro*. (**A**) CSCs migration induced by different concentration of SDF-1 for 12 h was detected with transwell migration assay. (**B**) CSCs migration induced by 100 ng/mL SDF-1 for different time was detected with transwell migration assay. (**C**) Representative images of migrated CSCs induced by 100 ng/mL SDF-1 with or without CXCR4 siRNA by transwell migration assay. 1: Medium alone group; 2: 100 ng/mL SDF-1 group; 3: CXCR4 siRNA group; 4: SDF-1 + CXCR4 siRNA group. (**D**) Representative images of migrated CSCs induced by 100 ng/mL SDF-1 with or without CXCR7 siRNA by transwell migration assay. 1: Medium alone group; 2: 100 ng/mL SDF-1 group; 3: CXCR7 siRNA group; 4: SDF-1 + CXCR7 siRNA group. Original magnification, ×100. Results were depicted as means ± SEM. *P < 0.05 versus control.

A transwell-based migration assay was established to quantitatively evaluate CSCs migration *in vitro*. As shown in Fig. 2A, compared with the control group, the average number of migrated CSCs increased significantly with 20, 50, 100 or 200 ng/mL SDF-1 induction, which reached a peak at 100 ng/mL. Furthermore, SDF-1-induced CSCs migration augmented in a time-dependent manner and reached its maximum at 24 h (Fig. 2B). Preincubated with CXCR4 or CXCR7 siRNA, SDF-1-induced CSCs migration was significantly suppressed (Fig. 2C,D). These data suggested that SDF-1 induced CSCs migration through CXCR4 and CXCR7.

### Effects of SDF-1, CXCR4 and CXCR7 on Akt, ERK and Raf-1 expression in CSCs

**Figure 3:**
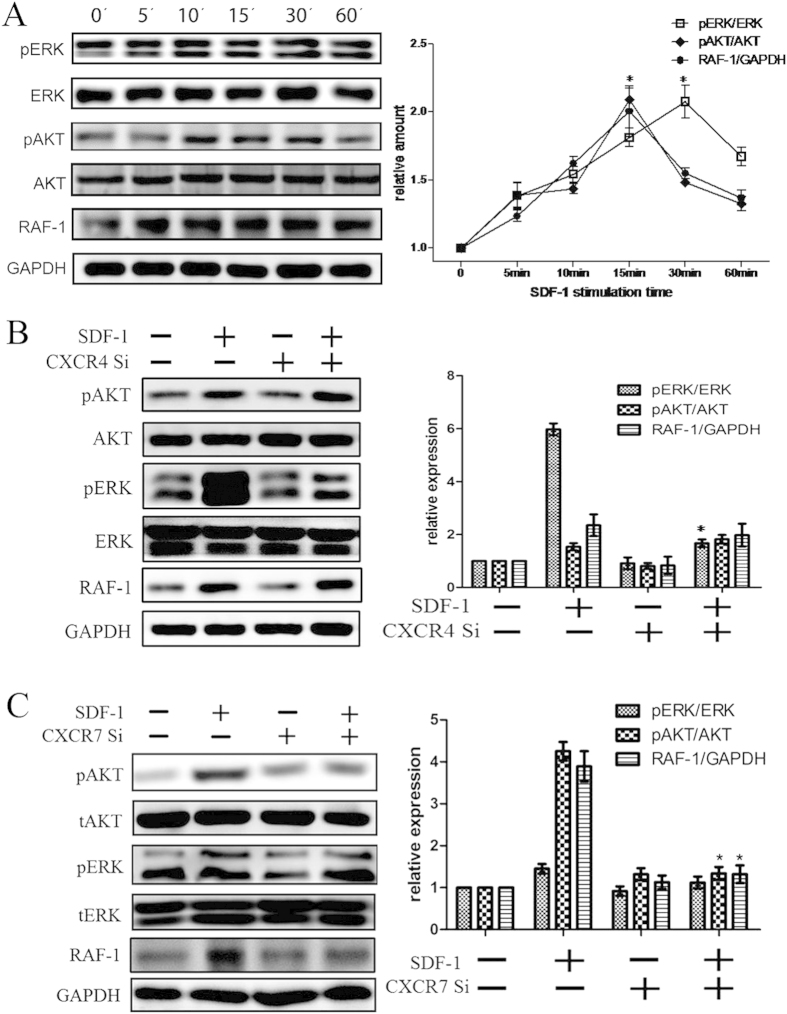
Effects of SDF-1, CXCR4 and CXCR7 on Akt, ERK and Raf-1 expression in CSCs. (**A**) Effect of SDF-1 on phospho-Akt, phospho-ERK and Raf-1 expression in CSCs was detected with western blot analysis. (**B**) Effect of CXCR4 siRNA on SDF-1-induced phospho-Akt, phospho-ERK and Raf-1 expression in CSCs was detected with western blot analysis. (**C**) Effect of CXCR7 siRNA on SDF-1-induced phospho-Akt, phospho-ERK and Raf-1 expression in CSCs was detected with western blot analysis. *P < 0.05 versus control.

After CSCs were treated with 100 ng/ml SDF-1 for different time (0, 5, 10, 15, 30, 60 min), phospho-Akt, phospho-ERK and Raf-1 proteins increased significantly in a time-dependent manner, which reached a peak at 15 min for phospho-Akt and Raf-1, and 30 min for phospho-ERK (Fig. 3A). With CXCR4 siRNA transfection, the expression of SDF-1-induced phospho-ERK decreased considerably, while SDF-1-induced phospho-Akt and Raf-1 expression did not change much (Fig. 3B). In contrast, with CXCR7 siRNA transfection, the expression of SDF-1-induced phospho-Akt and Raf-1 decreased considerably, while SDF-1-induced phospho-ERK did not change much (Fig. 3C). These results suggested that CXCR4 was involved in SDF-1-induced CSCs migration via MEK/ERK pathway, while CXCR7 was involved in it via PI3K/Akt pathway.

### Crosstalk between SDF-1/CXCR4 and SDF-1/CXCR7 pathway

**Figure 4:**
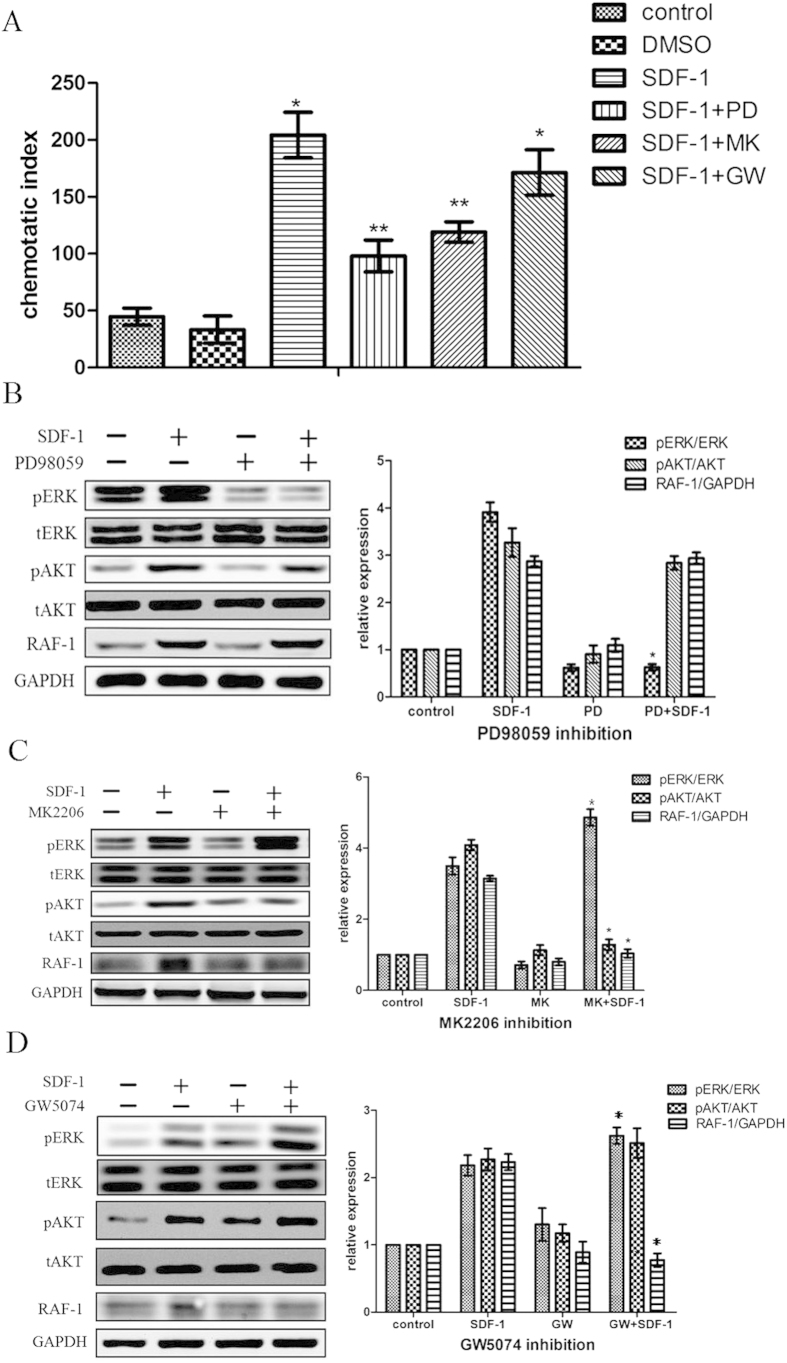
Crosstalk between Akt and ERK in CSCs migration. (**A**) CSCs migration induced by SDF-1 with different inhibitors was detected by transwell migration assay. *P < 0.05 versus control. **P < 0.05 versus SDF-1 group. (**B**) With ERK inhibitor PD98059, Akt, phospho-Akt, ERK, phospho-ERK and Raf-1 expression were detected by western blot. (**C**) With Akt inhibitor MK2206, Akt, phospho-Akt, ERK, phospho-ERK and Raf-1 expression were detected by western blot. (**D**) With RAF-1 inhibitor GW5074, Akt, phospho-Akt, ERK, phospho-ERK and Raf-1 expression were detected by western blot. *P < 0.05 versus SDF-1 group.

To determine whether there is a crosstalk between SDF-1/CXCR4 and SDF-1/CXCR7 pathway, we used different inhibitors to treat CSCs. As shown in Fig. 4A, by transwell migration assay, SDF-1-induced CSCs migration decreased markedly by pretreating CSCs with PD98059 (ERK inhibitor) or MK2206 (Akt inhibitor). However, GW5075 (Raf-1 inhibitor) did not suppress the migration of CSCs. Next, by western blot analysis, phospho-Akt, phospho-ERK and Raf-1 proteins were detected by pre-incubating CSCs with different inhibitors for 30 min. Results showed that ERK inhibitor PD98059 did not affect the expression of phospho-Akt and Raf-1 (Fig. 4B), whereas Akt inhibitor MK2206 down-regulated the expression of Raf-1 and up-regulated the expression of phospho-ERK (Fig. 4C). Besides, Raf-1 inhibitor GW5074 increased the expression of phospho-ERK, but had no effects on the expression of phospho-Akt (Fig. 4D). The above results indicated that Akt phosphorylation in SDF-1/CXCR7 pathway caused increase of Raf-1 expression, which in turn inhibited ERK phosphorylation in SDF-1/CXCR4 pathway and negatively regulated CSCs migration.

## Discussion

SDF-1 was revealed to be expressed in cardiac myocytes and fibroblasts, and MI significantly upregulates SDF-1^31^,^32^,^33^,^34^. Previous studies have revealed a protective role of SDF-1/CXCR4 signaling after MI and ischemia/reperfusion injury through survival effects on hypoxic cardiomyocytes^33^,^35^,^36^ and recruitment of CXCR4^+^ stem and progenitor cells^37^,^38^,^39^. However, it remains unclear whether SDF-1 play cardioprotectic roles through recruitment of CSCs to the infarcted area. The present study revealed that SDF-1 induced CSCs migration *in vitro* in a dose- and time-dependent manner, which could be suppressed by CXCR4 and CXCR7 siRNA.

CXCR4 and CXCR7 belong to the CXC subfamily and are involved in multiple physiological and pathological functions, such as cell survival, migration and metastasis^40^,^41^. Over the last few years, numerous studies have revealed the function of the SDF-1/CXCR4 axis during heart ischemia or after MI, whereas so far, the function of CXCR7 has been largely neglected. In contrast to CXCR4, CXCR7 exerts its biological effect mediated by β-arrestin, independently of G protein activation. Due to the absence of Gi-coupling, CXCR7 was initially regarded as a decoy receptor scavenging CXCL12 to prevent CXCR4 signaling and effects^42^,^43^. However, further investigations revealed that CXCR7 can activate Akt, MAPK, and JAK/STAT3 cascades, either by direct modulation, through a β-arrest independent pathway^26^,^27^,^28^,^29^, or after heterodimerization with CXCR4^18^,^20^,^28^,^30^.

In the present study, both CXCR4 and CXCR7 were detected in CSCs. Furthermore, SDF-1 induced increase of phospho-ERK and phospho-Akt expression in CSCs in a time-dependent manner. CXCR4 knockdown decreased the expression of phospho-ERK considerably, but did not change the expression of phospho-Akt and Raf-1. On the contrary, CXCR7 knockdown decreased the expression of phospho-Akt and Raf-1 considerably, but did not change the expression of phospho-ERK. These results suggested that CXCR4 was involved in SDF-1-induced CSCs migration via MEK/ERK pathway, while CXCR7 was involved in it via PI3K/Akt pathway.

Raf-1 is a serine/threonine kinase that can be activated by a variety of extracellular stimuli^44^. Activated Raf-1 proteins can phosphorylate and activate the MEK-ERK kinase pathway^45^,^46^. Crosstalk between the PI3K/Akt and the Raf/MEK/ERK pathways has been reported on multiple levels. Some studies suggest that the PI3K/Akt pathway enhances and/or synergizes with Raf/MEK/ERK signalling to provide a more robust signal. However, there is conflicting evidence that Akt directly phosphorylated Raf-1 on Ser-259 and resulted in a decrease in Raf-1 activity^47^,^48^,^49^,^50^,^51^. The inhibition of Raf-1 is due to the phosphorylation-dependent binding of the 14-3-3 protein, a negative regulator of Raf-1^51^. Thus, the crosstalk between Akt and Raf-1 depends on the type of ligand and the cellular background or stage of differentiation.

In the present study, results showed that ERK inhibitor PD98059 did not affect the expression of Raf-1 and phospho-Akt, whereas Akt inhibitor MK2206 down-regulated the expression of Raf-1 and up-regulated the expression of phospho-ERK. Besides, Raf-1 inhibitor GW5074 increased the expression of phospho-ERK, but had no effects on the expression of phospho-Akt. All these results indicated that Akt phosphorylation in SDF-1/CXCR7 pathway caused increase of Raf-1 expression, which in turn inhibited ERK phosphorylation in SDF-1/CXCR4 pathway and negatively regulated CSCs migration.

CXCR7 was shown to be involved in SDF-1-induced cell growth, survival and adhesion^52^,^53^, whereas its role in calcium mobilization and chemotaxis is still controversial^52^,^53^,^54^ and depends on the cell type. Liu *et al*.^55^ showed that CXCR4 was required for mesenchymal stem cell (MSC) migration and adhesion, whereas CXCR7 was responsible for MSC adhesion and survial. The molecular mechanisms which govern the responses remain unclear. The present study confirmed the chemotaxis of CXCR4 and CXCR7 on CSCs. Results showed that either CXCR4 or CXCR7 knockdown suppressed CSCs migration significantly. Although CXCR7 binds to SDF-1 with even higher affinity than CXCR4, a very distinct fine tuning of the down-stream signaling of both receptors may facilitate various cellular effects. CXCR7 has been shown to be a SDF-1 scavenger, inducing ligand internalization and degradation, which was thought to silence, inhibit, or regulate the functioning of CXCR4^42^,^43^. The present study also revealed that CXCR7 played an inhibiting role on CXCR4 in CSCs migration by modulating its signal transduction. Further studies are needed to clarify the detailed mechanisms involved.

In summary, our present study provided evidence that SDF-1 mediated CSCs migration through CXCR4 and CXCR7 via MEK/ERK and PI3K/Akt pathway, respectively. SDF-1/CXCR7/Akt pathway elevated Raf-1 expression and resulted in the inhibition of SDF-1/CXCR4/ERK pathway. The crosstalk between SDF-1/CXCR7 and SDF-1/CXCR4 pathway might provide us useful theories to repair infracted myocardium by mobilizing the CSCs.

## Methods

### Isolation and culture of CSCs

CSCs were isolated from the hearts of C57BL/6 rats as described previously^4^,^56^. Briefly, the C57BL/6 mice was sacrificed and the heart was rapidly excised. The heart was then cutted into several pieces and rinsed with PBS to remove blood. After that, the heart tissue was digested by 0.1% trypsin and 0.05% collagenase II for 5 min twice. Next, the heart tissue was planted and cultured in medium (Iscove’s Modified Dulbecco’s Medium with 10% fetal calf serum, 100 U/mL penicillin, and 100 U/mL streptomycin, 2 mmol/L L-glutamine,and 0.1 mmol/L 2-mercaptoethanol). Two or three weeks later, small phase-bright cells migrated from heart explants and were collected using CD117 PE Selection Kit (Stem Cell, Canada). These CSCs were cultured in CSCM (35% IMDM, 65% DMEM/F-12, 3.5% FBS, 2% B27, 0.1  mmol/L 2-mercaptoethanol, 10 ng/mL EGF, 20 ng/ml bFGF, 4 ng/mL Cardiotrophin-1, 1 U/mL thrombin, 100 U/mL penicillin, and 100 U/mL streptomycin,2 mmol/L L-glutamine) and were used for subsequent experiments. All procedures were performed in accordance with the Guidelines of the Hubei Council of Animal Care and approved by the Animal Use Subcommittee at the Huazhong University of Science and Technology, China.

### Cell transfection

CXCR4 and CXCR7 were knocked down using specific small interfering RNAs (siRNA) as previously described. For the knockdown of them, Stealth siRNA duplexes (Invitrogen) were transfected into the cells. Two different duplexes as well as negative control were used for CXCR4 and CXCR7. Cells were transfected at 50–70% confluence using RNAi MAX (Invitrogen) to give a final siRNA concentration of 20 nmol/L and were harvested 48 h after transfection.

### Transwell migration assay

Chemotaxis experiments were performed using a 24-well transwell chemotaxis chamber technique (Millipore, Billerica, MA, USA). Briefly, DMEM/F12 (600 μL) alone or medium containing 10, 20, 50,100 and 200 ng/mL recombinant rat SDF-1 (PeproTech, Rocky Hill, NJ, USA) was placed in the lower chamber. A total of 1 × 10^5^ CSCs in 200 μL medium were seeded into the upper chamber (pore size, 8 μm). For the inhibition experiment, CSCs were preincubated with 10 nmol/L CXCR7 siRNA or an inhibitor (20 μmol/L PD98059 for ERK, 20 μmol/L MK2206 for Akt, 50 μmol/L GW5074 for Raf-1) for 30 min prior to seeding. Then, CSCs and medium were transferred into the upper chamber. The chamber was then incubated for 12 h at 37 °C in a humidified atmosphere with 5% CO_2_. The membrane (Millipore) was removed and its upper surface was wiped away with a cotton swab to remove the unmigrated CSCs. The membrane was then fixed in neutral formalin for 10 min at room temperature and then stained with 0.1% crystal violet for 5 min. The number of CSCs that have migrated to the lower surface of the membrane was counted in 10 random high-power fields (HPFs) under a light microscope (Nikon Eclipse, Nikon Instruments, Inc., Melville, NY, USA). A chemotactic index (CI) was calculated to express stimulated migration: CI = stimulated migration (number of CSCs per HPF)/random migration (number of CSCs per HPF). Each assay was performed in triplicate wells.

### Western blot analysis

Whole cell extracts were prepared using commercially available RIPA lysis buffer (Thermo, MA, USA). Proteins (40 μg in total) were loaded on a 10% SDS-PAGE gel and transferred to NC membranes (Millipore, MA, USA) by semidry transfer. After blocking for 1 h with 5% (w/v) bovine serum albumin dissolved in 0.1% TBS-T, membranes were incubated overnight at 4 °C with the following primary antibody: anti-CXCR4 (ab2074, Abcam, Cambridge, UK) at 1/50 dilution, anti-CXCR7 (ab117836, Abcam, Cambridge, UK) at 1/50 dilution, anti-phospho-ERK1/2 (#4370, Cell signaling technology, MA, USA) at 1/2000 dilution, anti-ERK1/2 (#9102, Cell signaling technology, MA, USA) at 1/1000 dilution, anti-phospho-Akt (Ser473) (#4060, Cell signaling technology, MA, USA) at 1/2000 dilution, anti-Akt (pan) (#4691, Cell signaling technology, MA, USA) at 1/1000 dilution, anti-Raf-1 (ab18761, Abcam, Cambridge, UK) at 1/50 dilution, anti-GAPDH (#5174, Cell signaling technology, MA, USA) at 1/5000 dilution. After washing in 0.1% TBS-T, membranes were incubated with the appropriate HRP-linked secondary antibody for 1 h at room temperature. Detection of bound antibody was performed using the enhanced chemiluminescence kit (Pierce, Rockford, IL, USA). Bands were visualized using X-ray exposure machine (Genetech, Shanghai, China). All experiments were repeated 3 times (n = 3).

### RT- PCR analysis

Total RNA was isolated from CSCs using TRIzol reagent (Invitrogen, Life Technologies, USA) according to the manufacturer’s directions. Total RNA was then treated with DNase (Qiagen, Germany) and incubated at 65 °C for 30 min to remove any DNA contamination. The reverse transcription (RT) of total RNA was done with M-MuLV reverse transcriptase (Thermo Fisher, MA, USA) according to the manufacturer’s instructions. The cDNA generated by RT was then amplified by PCR using specific primers. The primers for CXCR4 were: (F) GACTGGCATAGTCGGCAATG, (R) AGAAGGGGAGTGTGATGACAAA. The primers for CXCR7 were: (F) AGCCTGGCAACTACTCTGACA, (R) GAAGCACGTTCTTGTTAGGCA. The primers for GAPDH were: (F) AGGTCGGTGTGAACGGATTTG, (R) TGTAGACCATGTAGTTGAGGTCA. PCR was performed in a programmable thermal cycler (ABI) with the following amplification profile: 30 sec at 94 °C followed by 30 cycles at 94 °C for 30 sec, 60 °C for 45 sec and 72 °C for 1 min. The samples amplified by PCR were visualized on a 1.0% agarose gel containing 0.001 mg/L Gold View (Tiangen, Beijing, China). All experiments were repeated 3 times (n = 3).

### Statistical analysis

All data are represented as the mean±SEM. Statistical analyses were performed using GraphPad Prism 5 (GraphPad Software, La Jolla, CA, USA). Data were analyzed using a one-way analysis of variance (ANOVA) followed by Tukey’s Honestly Significant Difference test. P < 0.05 was considered statistically significant.

## Additional Information

**How to cite this article**: Chen, D. *et al*. Crosstalk between SDF-1/CXCR4 and SDF-1/CXCR7 in cardiac stem cell migration. *Sci. Rep*. **5**, 16813; doi: 10.1038/srep16813 (2015).
