# Author Correction: Crosstalk between SDF-1/CXCR4 and SDF-1/CXCR7 in cardiac stem cell migration

**DOI:** 10.1038/s41598-022-06468-1

**Published:** 2022-02-11

**Authors:** Dong Chen, Yanli Xia, Ke Zuo, Ying Wang, Shiying Zhang, Dong Kuang, Yaqi Duan, Xia Zhao, Guoping Wang

**Affiliations:** Institute of Pathology, Tongji Hospital, Tongji Medical College, Huazhong University of Science and Technology, Wuhan, 430030 China

Correction to: *Scientific Reports* 10.1038/srep16813, published online 18 November 2015

This Article contains errors.

The images for the medium only and SDF-1 only groups are identical between Fig. 2C, D as the images in these figures were produced from the same experiment. This is not clearly stated in the article. For clarity, this figure is now corrected and the experiment is represented by a single panel; the legend of this figure is also revised to reflect this change. The corrected Fig. 2 is included below.

**Figure 2:**
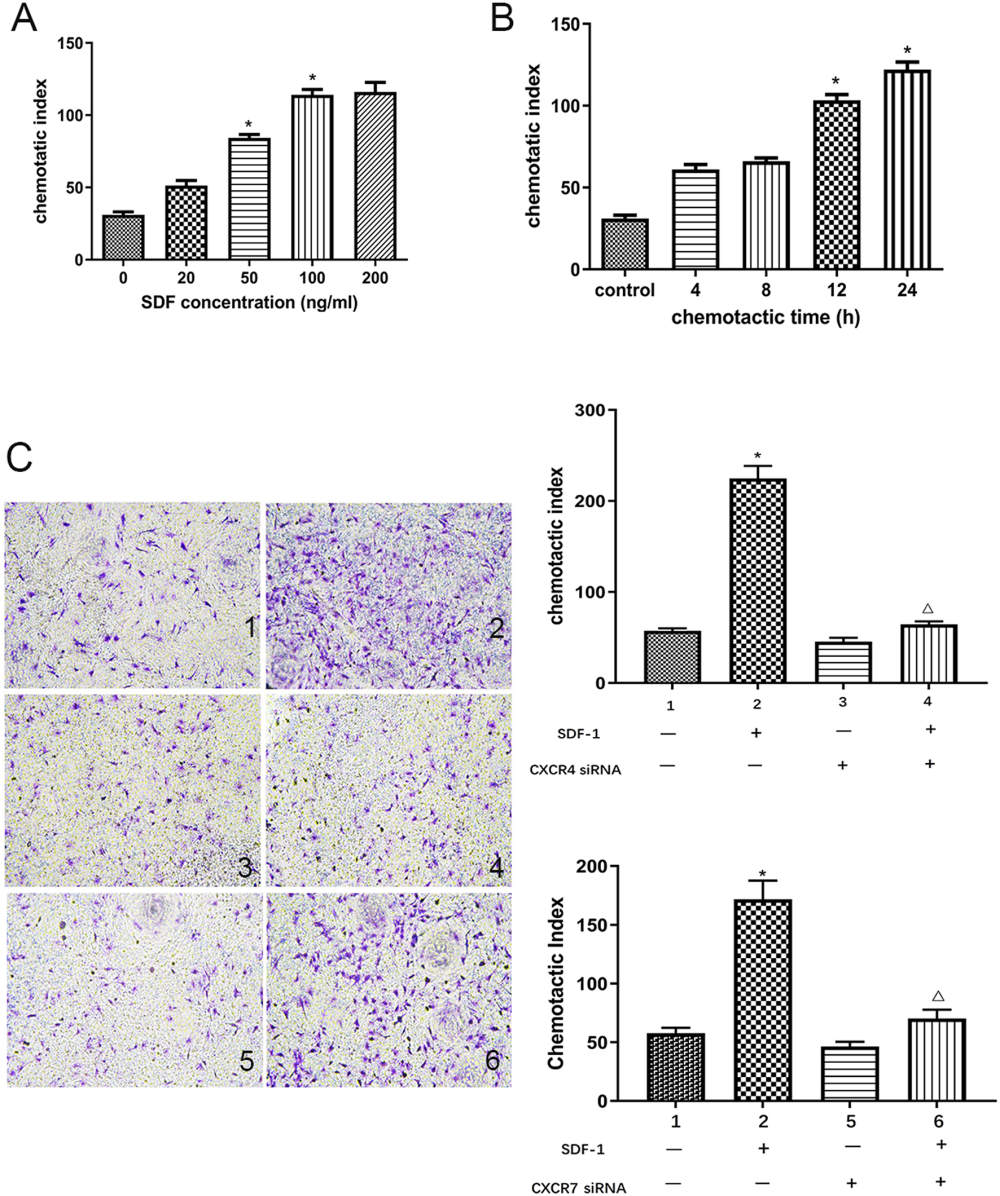
Effects of SDF-1, CXCR4 and CXCR7 on CSCs migration in vitro. (**A**) CSCs migration induced by different concentration of SDF-1 for 12 h was detected with transwell migration assay. (**B**) CSCs migration induced by 100 ng/mL SDF-1 for different time was detected with transwell migration assay. (**C**) Representative images of migrated CSCs induced by 100 ng/mL SDF-1 with or without CXCR4 siRNA or CXCR7 siRNA by transwell migration assay. 1: Medium alone group; 2: 100 ng/mL SDF-1 group; 3: CXCR4 siRNA group; 4: SDF-1 + CXCR4 siRNA group; 5: CXCR7 siRNA group; 6: SDF-1 + CXCR7 siRNA group. Original magnification, × 100. Results were depicted as means ± SEM. *P < 0.05 versus control, Δ*P* < 0.05 versus SDF-1 group.

Additionally, in the Methods section, ‘Transwell migration assay’,

"A chemotactic index (CI) was calculated to express stimulated migration: CI = stimulated migration (number of CSCs per HPF)/random migration (number of CSCs per HPF)."

should read:

“A chemotactic index (CI) was calculated to express stimulated migration:

$$ {\text{CI }}=\frac{\text {average number of migrated CSCs per HPF of experimental group or internal control}}{\text {average number of migrated CSCs per HPF of external control group}}\times 100"$$

These changes do not affect the conclusions of the Article.

